# Automatic COVID-19 severity assessment from HRV

**DOI:** 10.1038/s41598-023-28681-2

**Published:** 2023-01-31

**Authors:** Cosimo Aliani, Eva Rossi, Marco Luchini, Italo Calamai, Rossella Deodati, Rosario Spina, Piergiorgio Francia, Antonio Lanata, Leonardo Bocchi

**Affiliations:** 1 Department of Information Engineering, University of Florence, Florence, Italy; 2 UOs Anesthesiology and Reanimation Unit, San Giuseppe Hospital, Empoli, Italy

**Keywords:** Biomedical engineering, Vascular diseases

## Abstract

COVID-19 is known to be a cause of microvascular disease imputable to, for instance, the cytokine storm inflammatory response and the consequent blood coagulation. In this study, we propose a methodological approach for assessing the COVID-19 presence and severity based on Random Forest (RF) and Support Vector Machine (SVM) classifiers. Classifiers were applied to Heart Rate Variability (HRV) parameters extracted from photoplethysmographic (PPG) signals collected from healthy and COVID-19 affected subjects. The supervised classifiers were trained and tested on HRV parameters obtained from the PPG signals in a cohort of 50 healthy subjects and 93 COVID-19 affected subjects, divided into two groups, mild and moderate, based on the support of oxygen therapy and/or ventilation. The most informative feature set for every group’s comparison was determined with the Least Absolute Shrinkage and Selection Operator (LASSO) technique. Both RF and SVM classifiers showed a high accuracy percentage during groups’ comparisons. In particular, the RF classifier reached 94% of accuracy during the comparison between the healthy and minor severity COVID-19 group. Obtained results showed a strong capability of RF and SVM to discriminate between healthy subjects and COVID-19 patients and to differentiate the two different COVID-19 severity. The proposed method might be helpful for detecting, in a low-cost and fast fashion, the presence and severity of COVID-19 disease; moreover, these reasons make this method interesting as a starting point for future studies that aim to investigate its effectiveness as a possible screening method.

## Introduction

Coronavirus disease 2019 (COVID-19) is a very feared condition caused by Severe Acute Respiratory Syndrome Coronavirus 2 (SARS-CoV-2) infection^1^. SARS-CoV-2 is highly transmissible and pathogenic, for this reason COVID-19 was declared a pandemic by the World Health Organization (WHO) on March 11, 2020. The global mortality rate is about 3.4% (WHO, 2020), and observational studies indicate that preexisting conditions such as obesity, cardiovascular disease, diabetes, chronic respiratory disease, hypertension, and cancer increase this rate^2^. Efforts on the part of governments are focusing on promoting policies to contain the spread of the infection, and scientists are working to discover the etiological treatment of clinical manifestations from mild to very serious. However, because symptoms do not appear in the first phase of infection, self-isolation may begin too late. Thus, early indicators of COVID-19 are needed. COVID-19 disease typically arises with fever, cough, and dyspnea, and may lead to respiratory failure^3^. The minority of COVID-19 patients require treatment in the intensive care unit, but they can take a dangerous course due to a pathological inflammatory response called “Cytokine Storm”^4^. Many severe cases of COVID-19 are associated with progressive lung damage, which was partially attributed to the cytokine storm resulting in a loss of integrity of the capillary alveolus membrane causing edema, microvascular damage, and activation of coagulation^5^. Although the symptoms are predominantly respiratory, COVID-19 is not only a respiratory disease. Cardiac, renal, hemodynamic, and neurological manifestations are common in critically-ill COVID-19 patients derived from the pathogenesis of the virus on the vascular system^6–8^. A lot of different hypotheses (i.e. injury mediated by SARS-Cov-2 infection, hypoxia, excessive inflammatory responses) have been proposed with respect to the causes of cardiovascular damage that patients with COVID-19^8^. Among the direct effects of SARS-Cov-2 infection on the cardiovascular system in hospitalized patients there are acute heart failure, cardiogenic shock, ventricular dysfunction, and arrhythmias^9^. These conditions were associated with mortality and ICU admission^10^. In this sense, recent meta-analyzes showed that the prevalence of cardiovascular diseases among these patients such as acute heart damage and hypertension affect about 50% of patients^10^. Moreover, over 7% of COVID-19 patients have been reported to have myocardial damage. This percentage can rise to 22% in the case of critically ill patients^11^. Endothelial damage can also contribute to damage to the myocardium in addition to affecting the vascular system^8^. In particular, patients assisted in intensive care units often show microcirculatory alterations which appear to be related to the severity of the disease manifestation^12^. Given the strong relationship between cardiac and pulmonary activity^13–16^, this study focused on the relationship between COVID-19 and cardiovascular alterations. Specifically, the heart rate variability (HRV) dynamic has been analyzed to detect whether there could be differences in patients affected with different COVID-19 severity. In particular, in this study, HRV time series have been extracted from photoplethysmography (PPG) signals.

### HRV

Heart rate variability (HRV) represents the variation in time between successive heartbeats and correlates with complex nonlinear cardiovascular responses, due to autonomic nervous system patterns within critical illness^17–19^. HRV complex dynamic features indicate early cardiorespiratory complications^20,21^, autonomic dysfunction^22^, sepsis^23^ and death^24^. The latest research findings have shown the strength of HRV analysis in the extraction of a wide variety of traditional linear time and frequency domain features through more complex linear models that include additional physiological parameters such as respiration, arterial blood pressure, and intravascular body volume. Recently, through HRV analysis, nonlinear components of many physiological processes have been addressed^15,25^. A review of HRV extraction methods from different devices highlighted that although ECG devices have served as the gold standard, several alternative devices are more practical for extracting HRV time series^26^, mainly based on single-lead ECG and PPG. In another study, the comparison between HRVs from ECG and PPG showed that PPG data were accurate enough to detect cardiac rhythm alterations^27^. Despite the countless advantages of PPG devices, such as low cost, non-invasiveness, and easy applicability, PPG suffers from noise due to several signal artifacts that make it difficult to be validated as a monitoring system. These sources of artifacts can originate from inter-individual (skin tone, Body Mass Index (BMI), gender), physiological (respiration, measurement of body size, body temperature), and environmental (movements, ambient light, the pressure exerted on the skin) variability. These sources of variability, therefore, have limited the applications of pulse oximetry and the study of its derived functions.

### HRV and COVID-19

A number of other aspects combine to make the use of HRV interesting for the management of patients such as those affected by COVID-19. HRV has long been identified as a surrogate measure of cardiac autonomic tone^28^. Since HRV measures normal-to-normal (NN) interbeat interval (IBI) variations, it reflects complex interplays among physiological processes such as feedback, intrinsic mechanisms of pacemaker cells, thermogenesis, and the parasympathetic and sympathetic tone^29^. The Autonomic Nervous System (ANS) analysis could warn of an impending cytokine storm sooner than other currently employed laboratory tests thanks to its sensitivity to the measurement of changes in physiological states^4^. Earlier recognition of clinical deterioration could probably improve the chance of positive outcomes by triggering promptly therapeutic interventions. A novel meta-analysis of 51 studies demonstrated an inverse relationship between indices of HRV and markers of inflammation^30^. Earlier diagnosis of COVID-19 may be facilitated by studying heart rate variability in these patients. HRV parameters could not only help to detect COVID-19 promptly but could also help to identify patients at major risk of developing complications and assess the course of the disease^2^. In a previous work^31^, we had already studied the relationship between COVID-19 and HRV parameters on the same dataset of this work, adopting a purely statistical approach based on Mann–Whitney U test (MWUT), demonstrating the existence of statistical differences between some parameters of different groups. In this study, we used a new method of analysis and selection of features, and subsequently, a machine learning approach was implemented to achieve an automatic discrimination between groups. Therefore, as SARS-CoV-2 has been found to interact and affect the cardiovascular system and this interaction leads to microvascular dysfunction, the aim of this study was to investigate the relationship between COVID-19 and cardiovascular alterations using HRV parameters, extracted from a PPG signal. Specifically, HRV time series have been analyzed to detect alterations in patients with different severity of COVID-19.

## Methods

### Subject recruitment and physiological signal acquisition

In a total of 143 subjects, 93 patients with COVID-19 and 50 healthy controls, photoplethysmography (PPG) signals were acquired in this study. The patients were hospitalized patients at San Giuseppe Hospital (Empoli–Italy), while healthy subjects were healthcare operators working in the same hospital. Healthy subjects with preexisting health issues that could affect microcirculatory health status were not included in the study. Controls and patients were divided into three groups (Groups 0, 1, 2) based on the clinical condition. Controls were included in Group 0 (age = 45 ± 23, male/female ratio = 0.47) while patients were divided into two groups based on clinical condition: Group 1, patients with COVID-19 mild severity (age = 70 ± 15, male/female ratio = 1.61) and Group 2, patients with COVID-19 moderate severity (age = 66 ± 13, male/female ratio = 2.5). The protocol and the consent forms were approved by the Ethics Committee: Comitato Etico di Area Vasta Centro (CEAVC), protocol number: CEAVC19059. The study was performed according to the principles expressed in the Declaration of Helsinki^32^ and the informed consent was obtained from all subjects.

Photoplethysmography (PPG) signals were acquired through a commercial monitoring system. The system was composed of three parts: a finger pulse oximeter (connected to the SpO2 monitor), a touchscreen monitor (Mindray ePM 10^33^), and a data-logger to save data (Raspberry Pi 3). Each subject enrolled in the study underwent an acquisition protocol composed of two phases: acclimatization and acquisition. The acclimatization phase lasted 10 minutes necessary to reach a stable body temperature and avoid data bias. The acquisition phase lasted 5 min during which PPGs were acquired for all three groups of subjects by positioning the oximeter on the right forefinger.

### Clinical evaluation

The clinical classification of patients in group 1 (mild severity) and group 2 (moderate severity) was carried out based on the support of oxygen therapy and/or ventilation, necessary for the type of respiratory failure caused by COVID-19 pneumonia. Specifically, two indexes have been adopted.

The first was the Horowitz index, an index of lung function and of the respiratory system’s effectiveness to maintain the respiratory exchanges of the organism. This index measures the ratio between the partial pressure of oxygen in the arterial blood (PaO2-P) and the fraction of oxygen administered to the patient (FiO2-F).

The second index was the ROX index (i.e., [SpO2/FiO2]/respiratory rate). This index has been widely used during the SARS-CoV-19 pandemic in predicting the outcome of patients treated with HFNC and therefore is an index failure of oxygen therapy.

Specifically, the two patients groups were defined as follows:

- Group 1 was characterized by patients who performed only low-flow oxygen therapy (nasal cannula, face masks) or high-flow (HFNC—high flux nasal cannula) without positive pressure ventilatory support. They were patients with $P/F>200$ and $RR<20$ a/min;
- Group 2 was characterized by patients who received non-invasive positive pressure ventilatory support (CPAP- Continuous Positive Airway Pressure or NIV-Non invasive ventilation). These were patients who despite maximal oxygen therapy have $P/F <200$ and/or $pH<7.35$, $pCO2>48$ mmHg and/or $RR>20$ a/min.

### Signal processing and features extraction

**Figure 1:**
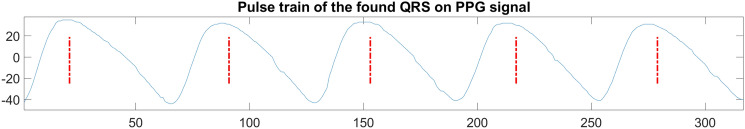
Perfusion peaks of a PPG signal extracted with the PT algorithm (red dashed lines).

All acquired signals were processed through algorithms implemented in Matlab (The Math Works, Inc., 2021). The first processing step was to identify and extract the time instants relating to the blood perfusion peaks. To this end, a modified version of Pan-Tompkins (PT) algorithm was applied. Generally, the PT algorithm is used to identify QRS complexes in electrocardiograms (ECG) to identify the time instants at which the R-peak occurs but it can also be used, properly modified, to identify systolic peaks of PPG signals^34,35^. In this study, the PT algorithm was modified to detect and extract the perfusion peaks of the PPG signals (see Fig. 1). In particular, low and high pass filter normalized cut-off frequencies were adjusted to PPG signal characteristics, respectively 3/fs and 0.2/fs where fs is the sampling frequency of the signals, 60 Hz. Indeed, since PPG is smoother than the ECG signal, it is characterized by a lower frequency bandwidth.

PT algorithm results were composed of a time series containing all the signal samples at which perfusion peaks occur, and it was converted into time domain series (in seconds) by dividing for the sampling frequency, obtaining a time series containing instantaneous PPG peaks (i-PPG). Eventually, a time series containing the temporal distances (or IBI: Inter-Beat Interval) between every temporal peak was obtained by the following equation:

1 $$\begin{aligned} IBI_{n} = (t_{p_{n+1}} - t_{p_{n}})\Big |_{n=1}^{N-1}, \end{aligned}$$

where $t_{p_n}$ is the *n*th-sample of the time series and N is the total number of temporal peaks of the signal.

Achieved time series were first analysed to remove noise artifacts. Furthermore, filtered signals were processed by Kubios software^36^ for HRV parameters extraction. For every IBI series, we computed a total of 43 parameters divided into three different domains: time domain, frequency domain, and non-linear parameters.

The time domain parameters are the following:

- *MEAN RR (ms)* the mean of RR intervals.
- *STD RR (ms)* standard deviation of RR intervals.
- *MEAN HR (beats/min)* the mean heart rate.
- *STD HR (beats/min)* standard deviation of HR.
- *RMDDS (ms)* square root of the mean squared differences between successive RR intervals.
- *NN50 (beats)* number of successive RR interval pairs that differ more than 50 ms.
- *pNN50 (%)* NN50 divided by the total number of RR intervals.
- *HRV TRIANGULAR INDEX (—)* the integral of the RR interval histogram divided by the height of the histogram.
- *TINN (ms)* baseline width of the RR interval histogram.

The frequency domain parameters are divided into different sub-domains based on frequency bandwidth:

- VLF (Very Low Frequency) includes frequencies in the bandwidth $[0.015 \div 0.04]$ Hz. This parameter is influenced, in a small part, by the Sympathetic Nervous System (SNS) and from changes in thermoregulation^17^;
  - *Peak (Hz)* VLF band peak frequency;
  - *Power (ms2)* absolute power of VLF band;
  - *Power (%)* relative power of VLF band.
- LF (Low Frequency) includes frequencies in the bandwidth $[0.04 \div 0.15]$ Hz. This parameter is influenced principally by the SNS, in small part from the Parasympathetic Nervous System (PNS) and the baroreceptors’ regulation activity^17^.
  - *Peak (Hz)* LF band peak frequency;
  - *Power (ms2)* absolute power of LF band;
  - *Power (%)* relative power of LF band;
  - *Power (n.u.)* power of LF band in normalised units.
- HF (High Frequency) includes frequencies in the bandwidth $[0.15 \div 0.4]$ Hz. This parameter is influenced principally by the PNS and vagal system and is also influenced by the breathing depth^17^;
  - *Peak (Hz)* HF band peak frequency;
  - *Power (ms2)* absolute power of HF band;
  - *Power (%)* relative power of HF band;
  - *Power (n.u.)* the power of HF band in normalised units.
- The ratio between Low-Frequency Power and High-Frequency Power is generally associated with the relationship between the sympathetic and parasympathetic system^17^.
  - LF/HF Power.

Non-linear parameters are listed as follows:

- *ApEn* approximate entropy;
- *SampEn* sample entropy;
- *MSE* multiscale entropy for scale factor values $\tau = 1, 2,..., 20$.

### Feature selection and machine learning algorithm

This paragraph reports on the methodologies used to determine the most significant feature set along with the machine learning algorithms implemented to automatically recognize COVID-19 severity. First, a statistical analysis was applied to the feature sets for investigating if there were parameters significantly different among Groups 0, 1 and 2. Those features were consequently helpful for the deep characterization of the groups.

Feature analysis was performed according to the Least Absolute Shrinkage and Selection Operator (LASSO) technique. This technique is a feature selection method based on a regularization process that aims to improve the accuracy of the prediction and the interpretability of the statistical model. Specifically, the regularization process removes those features without a decisive ability in representing the dataset, avoiding the model overfitting. The LASSO technique was adjusted for our dataset through the $\lambda$ parameter, which quantifies the features’ restriction. Of note, when $\lambda$ is 0, no features are removed from the dataset, and the LASSO collapses to the linear regression technique. Otherwise, when $\lambda$ increases, more features get removed from the dataset due to the more dataset representability requested to the features. Generally, the LASSO algorithm automatically employs a series of $\lambda$ values from 0 to $\lambda _{max}$, where the representability of the dataset is so high that all the features are removed. For every $\lambda$ value, the Mean Squared Error between the dataset and the computed model, with the features saved by that regularization process, was evaluated. Eventually, the best $\lambda$ value (i.e., $\lambda _{optimal}$), which minimizes the mean squared error, was calculated.

The results of features analysis allowed for identifying those parameters necessary for training two supervised classifiers aiming to automatically discriminate between healthy subjects (Group 0) and patients affected by COVID-19 with different severity (Group 1 and 2) using only HRV features.

In this study, two different classifiers, Support Vector Machine (SVM) and Random Forest (RF), were implemented to find which had the greater ability to discriminate between classes. In particular, all machine learning algorithms were implemented in the Matlab environment (The Math Works, Inc., 2021).

For the classification process, the Leave One Subject Out (LOSO) method was implemented. The LOSO technique consists in removing a subject from the dataset (composed of N subjects), training the classifier on N-1 subjects and testing it on the removed subject. Then, the removed subject is reinserted in the dataset, and the steps are repeated iteratively unless all the N subjects are removed at most one time. The global performance of the classifier has been evaluated by averaging the performance of every single iteration.

## Results

The application of the LASSO technique allowed to emphasize which HRV parameters were the most representative during comparisons between classes, respectively healthy subjects (Group 0), mild COVID-19 patients (Group 1), and moderate COVID-19 patients (Group 2). These parameters are shown in Table 1 while Table 2 reports the $\lambda _{optimal}$ and Mean Squared Error values for each group comparison. The features that are selected through the LASSO technique, are used as input parameters to train and test two different supervised classifiers, SVM and RF, in order to automatically detect both the presence of COVID-19 and its severity. The results of the SVM and RF classifiers for the two-group and three-group comparisons are shown in Tables 3 and 4, respectively. The obtained results show a high ability, particularly with the RF classifier, in the distinction between Group 0 and Group 1 with an accuracy of 94%, while in the comparison between Group 1 and 2 an accuracy of 89% was achieved. Since the comparison in Table 4 involves three different groups, only the classifiers overall accuracy parameter was evaluated. In particular, the overall accuracy of the RF classifier is 85% while the overall accuracy of the classification with the SVM classifier is 74%.

**Table 1:** Features retained from the LASSO technique for each group comparison.

Time domain	0/1	0/2	1/2	0/1/2	Entropies	0/1	0/2	1/2	0/1/2
Mean RR					ApEn	x		x	x
Std RR					SampEn				
Mean HR					MSE1				
Std HR	x				MSE2				
RMDDS					MSE3	x			
NN50					MSE4				x
pNN50	x			x	MSE5	x			
HRV Tri Ind	x				MSE6				
TINN					MSE7		x		x
Frequency domain	0/1	0/2	1/2	0/1/2	MSE8	x			
VLF Peak					MSE9				
VLF Power (ms$^2$)	x			x	MSE10	x		x	
VLF Power (%)					MSE11	x	x		x
LF Peak	x		x		MSE12	x		x	
LF Power (ms$^2$)					MSE13	x		x	
LF Power (%)	x	x	x	x	MSE14				
LF Power (n.u.)					MSE15		x	x	x
HF Peak	x	x		x	MSE16	x		x	
HF Power (ms$^2$)	x			x	MSE17	x	x	x	x
HF Power (%)					MSE18	x	x		x
HF Power (n.u.)					MSE19	x		x	
LF/HF Power					MSE20				x

**Table 2:** Optimal lambda ($\lambda _{optimal}$) and Mean Squared Error for each group comparison.

	0/1	0/2	1/2	0/1/2
$\lambda _{optimal}$	0.0184	0.0985	0.0511	0.0365
Mean Squared Error	0.1291	0.4020	0.2081	0.2969

**Table 3:** Classification results for each two-groups comparison with the two tested classifiers.

Test	Classifier	Specificity (%)	Sensibility (%)	Accuracy (%)
0 vs 1	RF	94	94	94
	SVM	88	91	90
0 vs 2	RF	92	93	93
	SVM	90	93	92
1 vs 2	RF	91	87	89
	SVM	77	78	77

**Table 4:** Classification results, expressed as a confusion matrix, for the three-groups comparison with the two tested classifiers.

Classifier		Class 0	Class 1	Class 2
RF	Class 0	46	3	1
	Class 1	7	36	4
	Class 2	3	3	40
SVM	Class 0	38	11	1
	Class 1	7	34	6
	Class 2	2	10	34

**Table 5:** Classification results for each two-groups comparison with the two tested classifiers, using only the LF Power (%) and MSE17 features.

Test	Classifier	Specificity (%)	Sensibility (%)	Accuracy (%)
0 vs 1	RF	86	87	87
	SVM	80	87	84
0 vs 2	RF	88	85	86
	SVM	88	89	89
1 vs 2	RF	81	83	82
	SVM	85	83	84

**Table 6:** Classification results, expressed as a confusion matrix, for the three-groups comparison with the two tested classifiers, using only the LF Power (%) and MSE17 features.

Classifier		Class 0	Class 1	Class 2
RF	Class 0	40	4	6
	Class 1	4	37	6
	Class 2	5	5	36
SVM	Class 0	38	10	2
	Class 1	6	34	7
	Class 2	2	6	38

**Figure 2:**
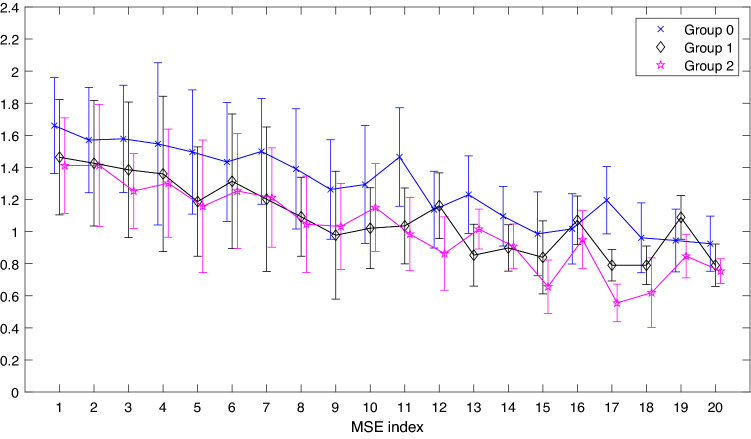
Multiscale Sample Entropy (MSE) for each value of scale factor $\tau$ = 1:20 and for each group. The symbols represent the median value for the three groups ($\times$ Group 0, $\diamond$ Group 1 and $\star$ Group 2). Whiskers represent the MAD value of the relative MSE value.

**Figure 3:**
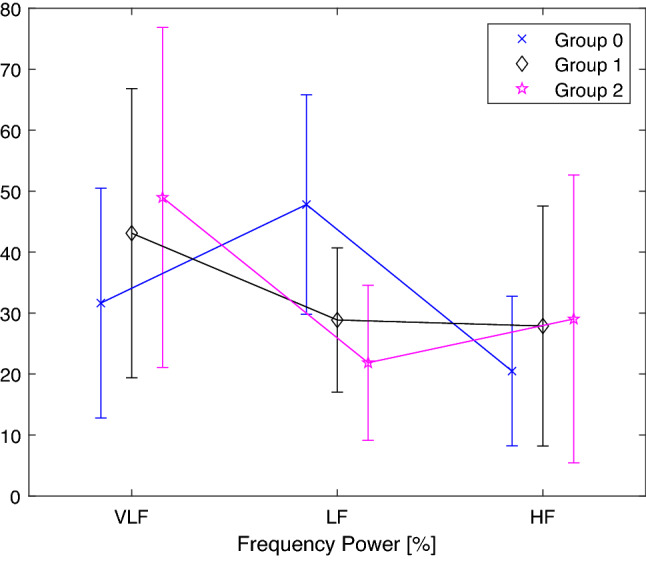
Frequency power (%) for each frequency band (VLF, LF and HF) and for each group. The symbols represent the mean value ($\times$ Group 0, $\diamond$ Group 1 and $\star$ Group 2). Whiskers represent the standard deviation value of the relative Frequency Power value.

As we can see from Table 1, the features that are most representative during the various comparisons are the frequency domain parameters and the entropy parameters. In particular, LF Power (%) and MSE17 are the only two features retained in each of the performed comparisons, both pairwise and in three-group comparisons suggesting that these features had a high capability of representing our dataset. Therefore, for a better analysis, the variation of MSEs and Frequency Power values between groups has been respectively highlighted in Figs. 2 and 3.

Figure 2 reports the trend of the median and respective mean absolute deviation (MAD) of each of the 20 MSE for each group (Group 0, Group 1 and Group 2). From a qualitative visual inspection, the figure shows that Group 0 (curve colored in blue within the figure) is almost always greater than Groups 1 and 2 (curves in black and pink), it suggests that MultiScale Entropies (MSEs) could differentiate the healthy from COVID-19 condition. For most of the parameters, it seems that a greater distance between the parameters’ median values corresponds to those MSE maintained by the LASSO feature selection method. Moreover, even though some parameters show closer median values, for example for MSE4 in Groups 1 and 2, yet they are maintained during the three-group comparison. This could be due to the fact that during pairwise comparisons the medians and MADs of MSE4 do not assume values that justify their maintenance, while, during a three-group comparison the introduction of the third group adds an informative content that changes classes relationship, thus enabling their distinction and maintenance of the parameter by the LASSO technique. It is worthwhile noting that the number of retained MSEs increases considerably for values of the scale factor $\tau>$ 10. As previously reported, for scale factor $\tau = 17$, MSE17 values were retained in each comparison. This fact is also confirmed by the relative position of the medians and MADs in Fig. 2, where one can see that for this value the three groups are clearly separated and distinguishable from each other. This fact recurs, albeit with less intensity, for the parameters MSE11, MSE15 and MSE18, where the corresponding parameter is maintained in three of the four comparisons between groups.

Figure 3 shows the trend of the mean and the standard deviation of the Power $(\%)$ parameter in the three frequency bands (VLF, LF and HF). The trend of these parameters suggests that LF Power (%) is representative of each of the classes and it is directly reflected in the Fig. 3 where this parameter is clearly different among the groups.

The representativeness of MSE17 and LF Power (%) can also be found when they are employed as training parameters for the SVM and RF classifiers. In fact, it is evident that when the classifiers are trained using only the two common parameters, the results of classification are extremely high (see Tables 5, 6). Again, since the comparison in Table 6 involves three different groups, only the classifiers with higher overall accuracy parameter were evaluated. In particular, the overall accuracy of the RF classifier is 79% while the overall accuracy of the classification with the SVM classifier is 77%. Results shown in Tables 5 and 6 are comparable to the results obtained when the training was carried out using all HRV parameters maintained from the LASSO features extraction method (see Tables 3, 4). In particular for the comparison between Group 0 and Group 2, the SVM classifier trained with all parameters achieves a specificity of 90%, sensitivity of 93% and accuracy of 92%, while the same classifier, trained only with LF Power and MSE17 parameters, reaches a specificity of 88%, sensitivity of 89% and accuracy of 89%, with an overall loss of accuracy of only 3%. Moreover, improved results are obtained during the comparison of the three groups, in fact (see Table 4) the use of an SVM classifier trained with all parameters achieves detection rates of 76% for Group 0, 72% for Group 1 and 74% for Group 2, on the other hand, the same classifier trained only with the LF Power (%) and MSE17 parameters reaches percentages of 76%, 72% and 83% (see Table 6) respectively, showing an improvement of 9% relative to Group 2.

The two parameters found to be common from the LASSO feature extraction operation could also have interesting medical findings. As mentioned earlier, LF band reflects both SNS activity, predominantly, and PNS activity. In literature, it is known how in presence of low respiration rate (< 7 breaths per minute) or deep breathing, LF becomes mainly an indicator of PNS so the LF values may be probably indicating an increased parasympathetic activity rather than an increase of sympathetic regulation^26^. Additionally, during periods of slow respiration rates, vagal activity can easily generate oscillations in the heart rhythms that cross over into the LF band^37–39^. Regarding the results obtained from the MSE analysis, we can see how, at the same time scale, the MSE values of healthy subjects are mostly greater than patients, indicating a greater signal complexity of the former. Another interesting aspect can be seen for the time scale $\tau = 5$, which, as reported by Costa et al. correspond the typical respiratory cycle length, for which there is a decrease in the value of the MSE for both groups of patients^40^. This could be due to the fact that the presence of COVID-19 precisely affects the respiratory system and its regulatory systems. Also, it can be seen that at the time scale $\tau = 17$ an extremely different information is highlighted in the three groups. As this is one of the scales with a higher index, this makes us hypothesize that its value is related to slow regulatory mechanisms, mechanisms that are precisely affected during COVID-19 infection.

## Conclusions and limitations

This study demonstrates an important ability to determine the presence of COVID-19, with different severity, using parameters of the HRV analysis performed on photoplethysmographic signals. In particular, with the LASSO technique is possible to select those features that are more characteristic for the discrimination between groups in each comparison. Obtained results show a very high classification accuracy achieved in each comparison, both between two groups and among three groups. The best result was obtained with the Random Forest classifier in discriminating on Group 0 and Group 1. In particular, it reached an accuracy of 94%, sensibility of 94% and specificity of 94%. Furthermore, it is worth noting that only two parameters are maintained in every LASSO feature selection: LF Power (%) and MSE17. This result led us to hypothesize that there could be a variation of the cardiovascular complexity expressed by the entropy measures depending on the COVID-19 severity. For this reason, the classification between groups was also performed using only these two parameters, obtaining excellent results. In particular, during the classification between Group 0 and Group 1, the RF classifier reached an accuracy of 87%, sensibility of 87% and specificity of 86%. The results of this study show that the use of only two HRV parameters: LF Power (%) and MSE17 may allow an excellent discrimination accuracy between healthy subjects and patients and between two groups of patients with different severity.

In our previous work^41^, we developed a method for detecting COVID-19 using a photoplethysmographic signal model. This method, specifically, used model parameters for COVID-19 detection and classification between healthy subjects and patients with moderate COVID-19. Considering that this is the same dataset and having tested the same classifiers, by comparing the two studies, it can be seen that in this study we achieved significantly better classification rates. This fact reinforces the power of the method and may also suggest a strong capability of HRV analysis in the analysis of COVID-19 pathology.

On the other hand, this study has some limitations related to the dataset, in particular, it’s known how circulatory parameters are affected by age and sex^42–45^. In our case, we did not take these two variables into account during the recruitment phase of all subjects, consequently, the difference in age and sex between the healthy group and the two patient groups requires that further studies are needed to better highlight these relationships and possibly also correlate them with the obtained result. Another limitation is the lack of knowledge of the patients’ state of health prior to COVID-19, even if we did not consider cases with severe COVID-19 but only mild and moderate.

## Data Availability

The dataset generated and analysed during the current study is not publicly available because informed consent is for participation in the study and not for third parties to see the complete data and analyze it, but is available from the corresponding author on reasonable request.
